# Antidiabetic Properties of Plant Secondary Metabolites

**DOI:** 10.3390/metabo13040513

**Published:** 2023-04-03

**Authors:** Stanislav Sukhikh, Olga Babich, Alexander Prosekov, Olga Kalashnikova, Svetlana Noskova, Alina Bakhtiyarova, Olesia Krol, Elena Tsvetkova, Svetlana Ivanova

**Affiliations:** 1 Institute of Living Systems, Immanuel Kant Baltic Federal University, A. Nevskogo Street 14, 236016 Kaliningrad, Russia; ssukhikh@kantiana.ru (S.S.); olich.43@mail.ru (O.B.); kalashnikova_14@bk.ru (O.K.); snoskova@kantiana.ru (S.N.); bakhtiarova.allina@yandex.ru (A.B.); ole-jolie@yandex.ru (O.K.); 2 Laboratory of Biocatalysis, Kemerovo State University, Krasnaya Street 6, 650043 Kemerovo, Russia; a.prosekov@inbox.ru; 3 Department of Biochemistry, St. Petersburg State University, 199034 Saint-Petersburg, Russia; evtsvetkova72@mail.ru; 4 Department of General Pathology and Pathological Physiology, Institute of Experimental Medicine, 197022 Saint-Petersburg, Russia; 5 Natural Nutraceutical Biotesting Laboratory, Kemerovo State University, Krasnaya Street 6, 650043 Kemerovo, Russia; 6 Department of TNSMD Theory and Methods, Kemerovo State University, Krasnaya Street 6, 650043 Kemerovo, Russia

**Keywords:** diabetes mellitus, plants, secondary metabolites, antidiabetic effect, insulin-like proteins, antioxidants

## Abstract

The prevalence of diabetes mellitus is one of the major medical problems that the modern world is currently facing. Type 1 and Type 2 diabetes mellitus both result in early disability and death, as well as serious social and financial problems. In some cases, synthetic drugs can be quite effective in the treatment of diabetes, though they have side effects. Plant-derived pharmacological substances are of particular interest. This review aims to study the antidiabetic properties of secondary plant metabolites. Existing review and research articles on the investigation of the antidiabetic properties of secondary plant metabolites, the methods of their isolation, and their use in diabetes mellitus, as well as separate articles that confirm the relevance of the topic and expand the understanding of the properties and mechanisms of action of plant metabolites, were analyzed for this review. The structure and properties of plants used for the treatment of diabetes mellitus, including plant antioxidants, polysaccharides, alkaloids, and insulin-like plant substances, as well as their antidiabetic properties and mechanisms for lowering blood sugar, are presented. The main advantages and disadvantages of using phytocomponents to treat diabetes are outlined. The types of complications of diabetes mellitus and the effects of medicinal plants and their phytocomponents on them are described. The effects of phytopreparations used to treat diabetes mellitus on the human gut microbiota are discussed. Plants with a general tonic effect, plants containing insulin-like substances, plants-purifiers, and plants rich in vitamins, organic acids, etc. have been shown to play an important role in the treatment of type 2 diabetes mellitus and the prevention of its complications.

## 1. Introduction

Diabetes mellitus (DM) is a dangerous and rapidly spreading disease [1]. In 2017, there were 425 million patients with a confirmed diagnosis of DM, and according to early projections, there will be 693 million cases worldwide by 2045 [2]. This spread is prompted by societal changes in nutrition and lifestyle. DM leads to serious cardiovascular complications, causing diabetic nephropathy, diabetic retinopathy, and neuropathy [3]. Diabetes can cause long-term damage, dysfunction, and failure of various organs, particularly the eyes, kidneys, nerves, heart, and blood vessels, in chronic conditions. Typical complications of diabetes are blindness (retinopathy), kidney failure, diabetic foot (severe foot damage that eventually leads to amputation), and cardiovascular disease [4].

The traditional classification divides DM into types 1 and 2, with type 2 diabetes accounting for the majority (>85%) of the overall incidence of diabetes mellitus [5].

The sudden onset of type 1 diabetes, which typically occurs in childhood or adolescence up to the age of 15, helps to accurately report new cases when prompt medical attention is sought. The top ten countries with a high prevalence of type 1 DM are the United States, India, Brazil, China, the United Kingdom, the Russian Federation, Algeria, Saudi Arabia, Nigeria, and Germany, which together account for almost 60% of all new cases. Countries in Europe and North America have a high to moderate incidence rate, while Africa has a medium incidence, and Asia has a low incidence, with the notable exception of Kuwait. The incidence of type 1 diabetes is increasing, averaging around 3.0% per year globally, with a peak during the cold season [6,7]. An increase in blood glucose levels is thought to be an adaptive measure in extremely cold climates that prevents the formation of ice crystals and acts as a cryoprotective mechanism for vital organs. Such adaptation, presumably, could have protected our northern European ancestors from climate change during the last ice age [8].

Type 1 DM is genetic in nature, but hereditary predisposition alone is not enough to cause this disease. The main role in its occurrence is played by environmental and lifestyle factors [6]. Type 1 DM can occur after pancreatitis or an organ transplant, during pregnancy, or as a result of cystic fibrosis. Insulin deficiency is absolute in type 1 diabetes, as evidenced by the autoimmune destruction of β-cells. Therapy for this type of diabetes involves exogenous insulin administration [9]. Type 2 DM has other causes such as β-cell deficiency, obesity, inflammation, insulin resistance, and chronic hyperglycemia [9]. Unlike type 1 DM, type 2 DM has an indolent development, so it is difficult to determine the exact dates of onset of the disease. Patients may experience elevated blood glucose levels over a long period of time (3–7 years), but there will be no clinical manifestations of the disease [6].

**Figure 1:**
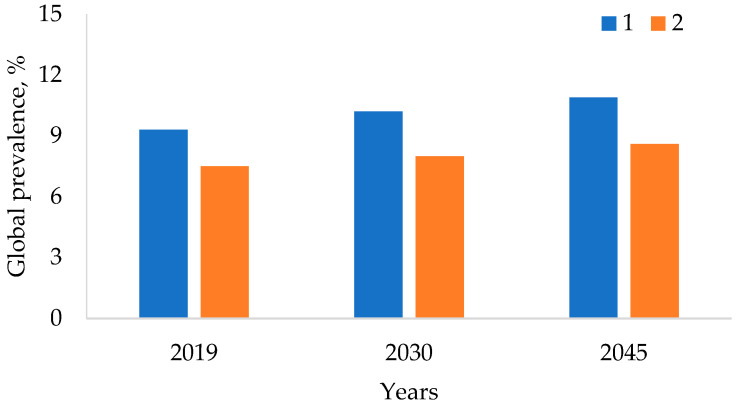
Global morbidity of impaired glucose tolerance: 1—diabetes; 2—insulin resistance.

Diabetes is estimated to have affected 9.3% (463 million people) of the global population in 2019, and is predicted to rise to 10.2% (578 million people) by 2030 and to 10.9% (700 million people) by 2045. One in every two (50.1%) diabetics is unaware of their condition (Figure 1). The global incidence of impaired glucose tolerance [10] was 7.5% (374 million cases) in 2019, rising to 8.0% (454 million cases) by 2030 and 8.6% (548 million cases) by 2045.

Diabetes and its complications have a significant economic impact on individuals and their families, as well as on the healthcare system and the national economy. Global direct healthcare costs due to diabetes were estimated at US$760 billion in 2019 and are expected to rise to a projected US$825 billion and US$845 billion by 2030 and 2045, respectively [10].

Currently, several synthetic medications (including sodium glucose cotransporter-2, metformin, thiazolidinediones, sulfonylureas, dipeptidyl peptidase 4 inhibitors, and glucagon-like peptide 1) are used to treat diabetes, all of which have side effects. For instance, thiazolidinedione and biguanide therapy can result in weight gain, and both of these medications have nephrotoxic effects. Incretin-based medications cause gastrointestinal problems [11].

Medicinal plants can be used as alternatives to synthetic medications in the treatment of DM. Their antidiabetic mechanisms include certain key targets such as α-glucosidase, α-amylase, dipeptidyl peptidase-4 (DPP-4), glitazone (PPAR γ), protein tyrosine phosphatase 1B (PTP1B), insulin-dependent protein–glucose transporter (GLUT4 (), etc. [12]. Additionally, some herbaceous plants improve hyperglycemia and insulin resistance through the adenosine monophosphate-activated protein kinase (AMPK) signaling pathway [12,13].

Xiao and Luo [14] have shown that traditional pharmaceutical therapy for T2DM is not effective enough to maintain satisfactory blood glucose levels in all patients. Furthermore, even patients who maintain a stable blood glucose level may experience secondary symptoms of type 2 diabetes and drug side effects. Traditional Chinese therapy with medicinal plant metabolites has shown promising results in T2DM clinical trials without causing the side effects associated with standard pharmaceuticals [14]. The economics of traditional Chinese medicine herbal diabetes treatment for patients and payers are often less than those of pharmaceutical regimens. Although several factors prevent traditional Chinese medicine from being fully assimilated into Western medical practices, it may be a viable alternative to treatment in contemporary healthcare settings [14].

Polyherbal mixtures known as “medical species” are used in traditional and officinal medicine [15]. This study examined medications used to treat diabetes mellitus and associated diseases in Russia. Medicinal species are mixtures of 2–15 plants, with mixtures of 3–6 plants being the most commonly mentioned in the literature. The antidiabetic activity of the main compounds identified in plants was predicted using the free PASS Online web resource. As a result, information on the composition, features of use, and dosage of 227 medicinal species was collected and analyzed.

It is shown in [16] that fucoidans are complex polysaccharides obtained from brown algae and consisting of a significant proportion of L-fucose and other monosaccharides, as well as sulfated ester residues. The search for new and natural bioproducts, dictated by the toxicity problems of chemotherapy drugs, has led to the extensive study of fucoidan due to reports of some of its biologically active characteristics. Among other bioactivities of fucoidan, antidiabetic and anticancer properties have attracted the most attention of researchers in the last decade. Research has shown that there is a link between diabetes and cancer; however, there are limited data on dual chemotherapeutic efforts. This study provides an overview of glucose metabolism, which is a central process involved in the progression of both diseases. Potential therapeutic targets were identified, and the relevance of fucoidan and its derivatives as a candidate for the treatment of both cancer and diabetes was demonstrated [16]. Antioxidant, anti-inflammatory, and anticoagulant medications based on fucoidan are used to treat COVID-19 and diabetes complications. Overall, fucoidan can be considered to be a promising candidate for the treatment of patients with COVID-19 and DM [17].

According to research by Oduro et al. [18], endothelial dysfunction, activation, inflammation, and permeability of the endothelial barrier are major causes of vascular complications in diabetes, such as cardiovascular disease. New avenues for comprehending the molecular mechanisms and therapeutic targets of endothelial dysfunction in diabetes have emerged as a result of the recent growth in molecular, transcriptional, and clinical research. Another review [18] discussed current critical and emerging molecular signaling pathways involved in the pathophysiology of endothelial dysfunction, as well as viable targets for pharmacological treatment. It is important to note that the polyherbal mixtures of traditional Chinese medicine (TCM) were studied and that biologically active compounds that modulate the effects of endothelial dysfunction in diabetes were isolated. Clinical study data on biomarkers and biochemical parameters involved in evaluating the effectiveness of the treatment of vascular endothelial dysfunction in diabetes were compared between clinically used Western hypoglycemic drugs and TCM formulas [18].

Natural herbal treatments for diabetes (more than 400 herbs can be found in the literature) are safe and efficient substitutes for Western medications, according to [19]. The focus of that study was on documenting the herbal medicines used by Sri Lankan Ayurvedic physicians and the amount of research undertaken with each of these herbal medicines [19].

The search for effective and safe bases of antidiabetic drugs among plants has been ongoing for more than a decade [20,21,22,23,24], but there is still no understanding of the mechanisms of antidiabetic activity formation and directions for targeted search. Any research that adds to our understanding in this area moves humanity closer to the possibility of curing and/or preventing diabetes mellitus.

Thus, the prevalence of diabetes mellitus is one of the major medical problems that the modern world currently face. Diabetes leads to early disability and death, as well as serious social and financial problems. Synthetic drugs are very effective in treating diabetes, but they have many side effects. Pharmacological substances derived from plants are of particular interest in the treatment and prevention of type 2 diabetes. This review sought to investigate the anti-diabetic properties of plant secondary metabolites.

This review examines plants and their metabolites and the techniques for extracting compounds from plants that are effective in the treatment of type 2 diabetes mellitus. Their use in treating this disease demonstrates the potential for their use in this field, confirms the topic’s relevance, and advances knowledge of the properties and mechanisms of action of plant metabolites. The review also discusses the main benefits and drawbacks of using phytocomponents in the treatment of diabetes mellitus and its complications.

## 2. Materials and Methods

The use of medicinal plants in the treatment of diabetes mellitus was examined using scientific publications and patents. The following keyword combinations were used in the search: diabetes mellitus, plants, secondary metabolites, antidiabetic effect, insulin-like proteins, and antioxidants. The search was conducted in the Scopus, Web of Science, PubMed, and Elibrary databases and in open internet sources from the early 1990s (appearance of the first publication on the topic) to the present. The emphasis was on articles published in peer-reviewed scientific journals with a high citation index in the previous five years. Generalization was the main method. Statistical and research data related to the study of various ways to reduce the negative impact of diabetes on animals and humans were analyzed, as were the medicinal plants and their constituents used in the treatment of diabetes mellitus.

## 3. Interaction of Intestinal Microbiota and Herbal Medicines in the Treatment of DM

Gut bacteria, also known as the gut microbiota, form a complex community of organisms that have a major impact on the health of the human body [25]. A growing number of researchers have focused on the role of the gut microbiota in disease prevention and treatment, including the relationship between gut microbiota, T2DM, and medicinal plants.

The microbiota and the human body share a common platform between cell signaling and defense against potential pathogens [26]. Bacterial taxonomy based on the phylogenetic diversity of ribosomal RNA or the 16S subunit of the rRNA gene allows for transcriptionally variable sequencing of a diverse range of microbial populations [27]. Different organisms can provide functional gene material, and deviations from this material are associated with various physiological states [28].

Most interactions between the human body and the gut microbiota occur through the gastrointestinal barrier. Both barriers (bacterial and/or mucosal) are important to prevent the movement of commensal bacteria, pathogens, and food antigens from the abdomen into the intestinal tissues and into the human systemic circulation [29]. Bacterial barriers serve many biological functions and are the first line of defense against bacteria. Gut microbes have a higher oxygen tolerance and catalase expression than fecal bacteria due to the oxidation gradient [30]. The membranes contain physical and chemical barriers that allow the gut microbiota to interact with the human body [31]. Physiology dysfunction leads to constipation, inflammation and the launch of enzymatic mechanisms and the immune response.

The induction of metabolic inflammation and the reduction of short-chain fatty acids by the gut microbiota are the two main mechanisms of interaction between the gut and human microbiota in type 2 diabetes. In 2010, significant differences were found in the composition of the gut microbiota between patients with type 2 diabetes and healthy controls. The rectal group is closely related to the concentration of glucose in the blood. Additionally, representatives of betaproteobacteria are often found in patients with type 2 diabetes [32]. With increasing scientific evidence, an imbalance between beneficial and harmful bacteria is believed to play an important role in the pathogenesis of type 2 diabetes. Increasing the number of short-chain fatty-acid-producing bacteria, especially butyrate-producing bacteria, and reducing the number of opportunistic microbes are fundamental approaches in the treatment of type 2 diabetes [33].

The pathogenesis of T2DM is closely associated with mild inflammatory conditions, also known as cytokine-induced acute phase reactions, and activation of the human immune response [34]. In the pathogenesis of type 2 diabetes mellitus, lipopolysaccharides secreted by Gram-negative bacteria can enter the intestinal circulation, disrupting the intestinal microenvironment and triggering an immune response in adipose tissue. TLR expression is upregulated and pro-inflammatory adipocytokines such as IL-1, IL-6, and TNF-α are released, resulting in a mild inflammatory state [35]. As key factors influencing cellular immunity, T cells play a critical role in the development of type 2 diabetes and associated inflammation. T cell metabolism is closely related to insulin and subsequent insulin receptor signaling, and the absence of insulin receptors on T cells inhibits glycolysis [36]. In addition, CD4+ T cells are associated with obesity and insulin resistance, which are major risk factors for type 2 diabetes [37]. Subtypes of CD4+ T cells, including Th1 and Th2 cells, when activated, can secrete a large number of pro-inflammatory cytokines to regulate the inflammatory process [38]. As evidence accumulates that the gut microbiota plays an important role in host immunity and subsequent inflammation, the important mechanisms of GM in the treatment of type 2 diabetes are becoming clearer. The therapeutic mechanism underlying its effectiveness is associated with its anti-inflammatory and immunomodulatory effects on the intestine.

The clinical use of herbal medicines for the treatment of T2DM is widespread throughout the world. However, due to the low bioavailability and bioactivity of herbal remedies after oral administration, there are still many gaps in understanding the possibility of using such drugs [39]. In recent years, it has been established that the intestinal microflora of the microbiota critically regulates the biotransformation of hypoglycemic herbal remedies, converting less polar lipoprotein complexes into more polar, lipid-rich compounds, thereby enhancing oral absorption and facilitating T2DM therapy. Phyto preparations can affect the intestinal microbiota, and, indirectly, on the recurrence of DM. Once metabolized by gut microbes, the bioactivity and bioavailability of the new compounds differ from the original herbal chemical. Under the influence of herbal preparations, intestinal bacteria can metabolize beneficial compounds. In this case, the composition may vary depending on the metabolic activity of different bacteria.

## 4. Main Plants Used to Treat Diabetes

This section presents the main plants that simultaneously exhibit high antioxidant and antidiabetic activity. Examples are representatives of classes of compounds with antioxidant activity that are used to treat diabetes and existing side effects [40,41,42,43,44,45].

### 4.1. Barberry (Berberis)

**Figure 2:**
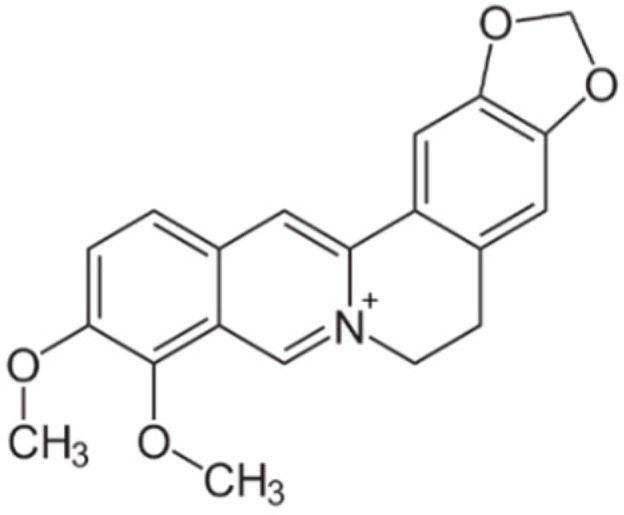
Chemical formula of berberine (C_20_H_17_NO_4_).

Barberry (family *Berberidaceae*) is found all over the world and is used as both a food and a spice. The isoquinoline alkaloid berberine has been isolated from barberry (Figure 2) and has been shown to be effective in the treatment of DM and other metabolic diseases [40]. In addition to DM, berberine is used in the treatment of cancer, digestive, cardiovascular and neurological diseases. Berberine can inhibit the growth of bacteria, in particular *Helicobacter pylori*. This alkaloid regulates glycometabolism and lipid metabolism, improves energy expenditure, reduces body weight, and alleviates nonalcoholic fatty liver disease. Berberine also improves cardiovascular hemodynamics, suppresses ischemic arrhythmias, and reduces the developments of atherosclerosis and hypertension. Berberine exhibits potent neuroprotective effects, including antioxidant, anti-apoptotic, and anti-ischemic effects [46].

However, a significant limitation of the use of berberine is its low bioavailability as a result of low intestinal absorption combined with its elimination by P-glycoprotein [47].

Zhang et al. have shown that berberine has an effect on the human gut microbiome that underlies the antidiabetic effect and that berberine therapy can cause more gastrointestinal side effects. The hypoglycemic effect of berberine is mediated by inhibition of DNA biotransformation by *Ruminococcus bromii* [48].

Berberine has also been shown to have a positive effect on preventing brain damage and improving cognitive function by reducing the risk of metabolic dysfunction and cardiovascular, kidney, and liver diseases [49].

Nanoparticles based on lecithin and chitosan filled with berberine were used for wound healing in a diabetic animal model. The optimized nanoparticle system reduces inflammation, stimulates the proliferation of blood vessels and fibroblasts, and promotes the deposition of mature collagen fibers [19]. Zhou et al. also showed that topical application of berberine accelerated diabetic wound healing by activating thioredoxin reductase 1 (TrxR1). This mechanism is a promising strategy for restoring redox homeostasis and accelerating the healing of diabetic wounds [50].

Berberine has also been shown to have a therapeutic effect in type 2 DM (improving insulin resistance, regulating glucose and lipid metabolism) through inhibition of the hypothalamic–pituitary–adrenal pathway axis [51].

### 4.2. Turmeric (Curcuma longa)

**Figure 3:**
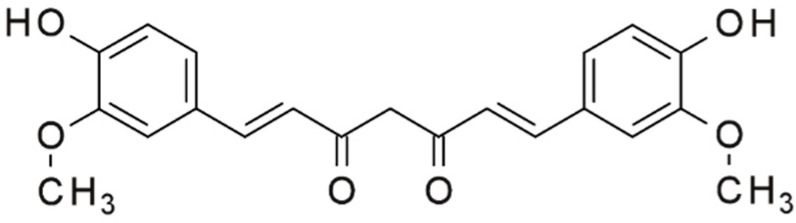
Chemical formula of curcumin.

Curcumin, a polyphenol isolated from turmeric root (Figure 3), is used as a coloring agent in the food industry. Curcumin is a biologically active molecule, a natural polyphenol present in the turmeric rhizome. Curcumin has various pharmacological and biological effects reported in in vitro and in vivo studies including antioxidant, cardioprotective, anti-inflammatory, antimicrobial, nephroprotective, antitumor, hepatoprotective, immunomodulatory, hypoglycemic, and antirheumatic effects. In animal models, curcumin extract delayed diabetes, improved β-cell function, prevented β-cell death, and reduced insulin resistance [52]. Studies have proven its therapeutic effect against DM [53]. For example, 1500 mg of curcumin taken on an empty stomach daily has been shown to reduce blood glucose levels and weight in type 2 diabetes patients [41].

Curcumin can suppress oxidative stress and inflammation. In addition, it significantly reduces fasting blood glucose, glycated hemoglobin, and body mass index [54]. Additionally, curcumin-based dietary supplements have a positive effect on blood lipids in prediabetes and type 2 DM [55,56].

Asadi et al. showed that curcumin supplementation for two months improved and reduced the severity of distal symmetrical polyneuropathy in patients with type 2 DM [57]. Curcumin intake may reduce the complications of diabetes by lowering triglyceride levels, as well as inflammation indicators of C-reactive protein and adiponectin [57]. Liposomal curcumin at a dose of 2 mg/0.1 kg of body weight showed an optimal therapeutic effect as a premedication for streptozotocin-induced DM [58].

### 4.3. Bitter melon (Momordica charantia)

*Momordica charantia* Linnaeus (*Cucurbitaceae*) has traditionally and widely been used as a food and herbal remedy for type 2 diabetes in Asia, Brazil and East Africa [59]. The hypoglycemic activity of a crude extract of *M. charantia* L. in rabbits sparked interest in the antidiabetic activity of *M. charantia* L. in the 1940s. More recently, several in vivo studies have shown the significant glucose lowering potential of whole fruits, fruit pulp, and seeds [60].

The therapeutic mechanisms of *M. charantia* focus on improving insulin resistance by increasing glucose uptake, consumption, and utilization [61].

**Figure 4:**
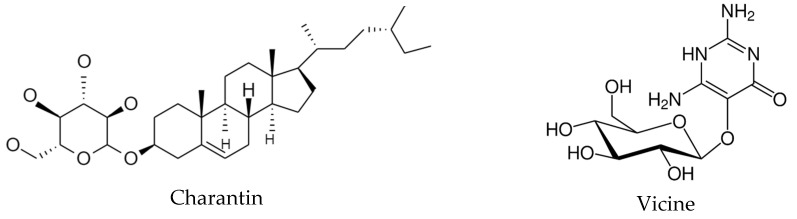
Active compounds of *Momordica charantia* L.

Compounds that have been isolated from *M. charantia*, including the insulin-like peptide charantin and the alkaloid vicine (Figure 4), have hypoglycemic effects. The largest amount of charantin is found in the pulp, while vicine is present in abundance in the fruit [62]. Furthermore, the active compounds in this plant increase pancreatic insulin secretion, decrease insulin resistance, and improve the ability of peripheral and skeletal muscle cells to use glucose. They also inhibit glucose absorption in the intestine and suppress key enzymes in gluconeogenic pathways [42].

Kim et al. showed that eating bitter melon reduced fasting blood glucose but did not reduce glycated hemoglobin. Serious adverse events during treatment were not recorded [63]. Bitter melon supplementation was shown to prevent diabetes in healthy rats. Bitter melon consumption reduced the rate of complications (polydipsia, polyuria, glucosuria, renal hypertrophy, and increased glomerular filtration rate) in diabetic rats. Melon consumption also resulted in a 31.64% decrease in blood glucose and a 27.35% increase in insulin in hyperglycemic rats [62].

*Lactobacillus plantarum* fermentation improves the antidiabetic properties of *M. charantia* juice by promoting gut microbiota regulation and the production of short chain fatty acids [64]. *M. charantia* improves pancreatic function by activating pancreatic beta cells, increasing insulin and Pdx1 gene expression, decreasing Glut2 expression and protecting liver tissue [65].

*M. charantia* saponins can normalize body weight, lower fasting blood glucose, improve insulin resistance, and increase hepatic phosphorylated adenosine monophosphate-activated protein kinase (p-AMPK)/total protein. Because of their antioxidant activity, the polysaccharides in this plant can repair streptomycin-damaged pancreatic β-cells [66].

The gastro-resistant peptide mcIRBP-9 from *Momordica charantia* induces modulation of blood glucose homeostasis in diabetic mice. In addition to controlling blood glucose and glycated hemoglobin levels, this peptide has anti-inflammatory and renoprotective properties [67]. Furthermore, extracts of this plant have hepato- and nephroprotective properties [68].

### 4.4. Ginseng (Panax)

Ginseng derivatives can be used in diabetes treatment [69]. Ginseng supplementation significantly reduced serum concentrations of fasting plasma glucose, total cholesterol, interleukin-6, and insulin resistance index, and also lowered tumor necrosis factor-α levels. Ginseng intake alters total and low-density lipoprotein cholesterol levels [70,71].

**Figure 5:**
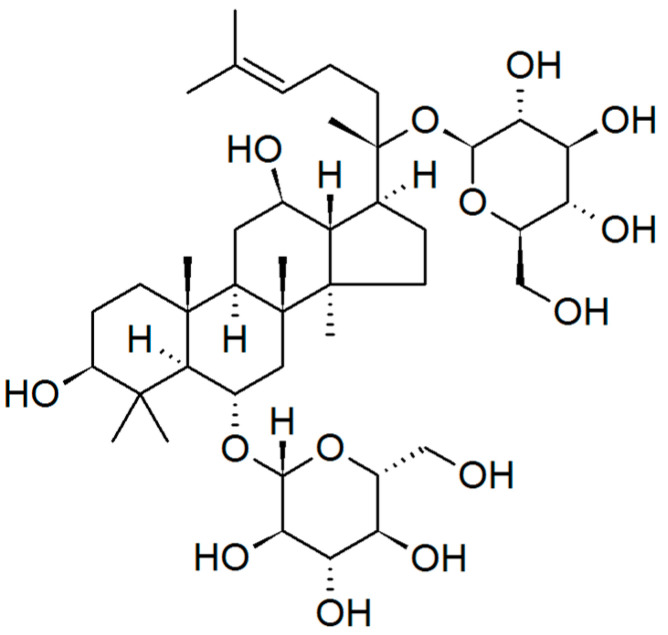
Ginseng ginsenoside, belonging to the class of natural steroid glycosides and triterpene saponins.

A combination of two widely consumed ginseng species (American ginseng and Korean red ginseng), enriched with ginsenoside (Figure 5), was shown to improve glycemic and blood pressure control in a study by Jovanovski et al. [43]. American ginseng may reduce postprandial glycemia by lowering glycated hemoglobin and glucose levels [72].

*Panax japonicus* can lower blood glucose levels in animals 1 h after administration of epinephrine hydrochloride. The active compounds of the plant responsible for hypoglycemic activity are ginsenosides and malonyl ginsenosides [73]. *Panax notoginseng* is used in traditional Chinese medicine for diabetic kidney disease, with a positive effect on lipid metabolism [74]. Preparations based on this plant protect kidneys from inflammatory damage by increasing autophagy through suppression of mTOR and activation of PINK1/Parkin signaling [75].

Ethanol extract and *Panax notoginseng* saponins alleviate oxidative stress in the vasculature in obese mice. The ethanol extract also increased glucose sensitivity and normalized blood pressure in diabetic mice [76]. In a study by Yuan et al., *Panax notoginseng* lowered albuminuria and the transition of epithelial to mesenchymal podocytes in diabetic rats, in part by inhibiting the Wnt/β-catenin signaling pathway [77].

### 4.5. Siberian Ginseng (Eleutherococcus)

The active compounds of *Eleutherococcus* that exhibit an antidiabetic effect are lignans, phenylpropanoids, flavonoids and triterpenes. The mechanisms of their therapeutic action include the inhibition of α-glucosidase and α-amylase and the improvement of insulin resistance [44,78,79]. However, there are very few studies that have investigated the hypoglycemic effects of *Eleutherococcus*.

### 4.6. Golden root (Rhodiola rosea L.)

**Figure 6:**
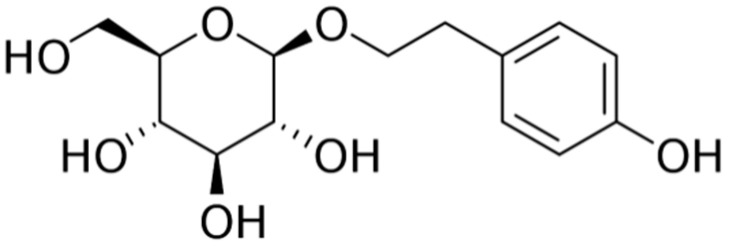
Structural formula of salidroside and tyrosol glucoside from *Rhodiola rosea* L.

Rhodiola species extracts are widely used as herbal medicines or dietary supplements in Asia, Europe, and the United States. Salidroside (Figure 6), a p-hydroxyphenethyl-β-glucoside compound, is the main active ingredient in Rhodiola root. Several studies have recently revealed that rhodiola and salidroside may have pharmacological properties that can be used to treat diabetes, with studies confirming that AMP-activated protein kinase (AMPK) and AMPK-associated signaling are associated with its beneficial effects [80].

*Rhodiola rosea* and its active compounds, comprising an important immunomodulator, exhibit a variety of pharmacological effects in various models of type 2 diabetes, including inhibition of hepatic gluconeogenesis, suppression of adipogenesis and lipid peroxidation, increased survival of islet B-cells, and so on, due to anti-inflammatory action.

There have been relatively few clinical studies on the treatment of diabetes with *Rhodiola rosea* L., and published results indicate that it is often used to treat diabetic nephropathy and retinopathy in the treatment of type 2 DM. However, experimental studies have shown that extracts or active compounds of *R. rosea* L. have a significant inhibitory effect on islet B cell apoptosis and an excessive inflammatory response, indicating the possibility of clinical treatment of DM 2 [81].

In diabetic rats, the salidroside from *R. rosea* L. showed hypoglycemic and anti-osteoporotic effects. An eight-week intake of salidroside dose-dependently reduced blood glucose, serum insulin, serum creatinine, blood urea nitrogen, and the level of albumin in the urine of rats with diabetic nephropathy and restored pathological changes in the renal tissue, renal sclerosis and fibrosis [82].

*Rhodiola rosea* has different chemical components, and different chemical elements have the same pharmacological effects and medicinal properties in various cardiovascular diseases caused by DM [83]. In addition, rhodiola has a nephroprotective effect [77]. *Rhodiola rosea* extract has a protective effect on early damage to alveolar bone in diabetic rats, and its mechanism is mainly the promotion of bone formation and the inhibition of bone resorption, as well as the balancing of bone metabolism [84].

*R. rosea* extract can activate insulin receptor substrate (IRS) and adenosine 5′-monophosphate (AMP)-activated protein kinase (AMPK) pathways to enhance insulin signaling, promote nutrient metabolism, and achieve the effect of improving *C. elegans* insulin resistance [45].

### 4.7. Stevia (Stevia rebaudiana Bertoni)

Stevia is a sweet natural glycoside and calorie-free sweetener extracted from the leaves of *Stevia rebaudiana* Bertoni and used as a substitute for artificial sweeteners [85]. Stevia also has antioxidant activity. The antidiabetic properties of stevia are due to the inhibition of advanced glycation end products [86].

**Figure 7:**
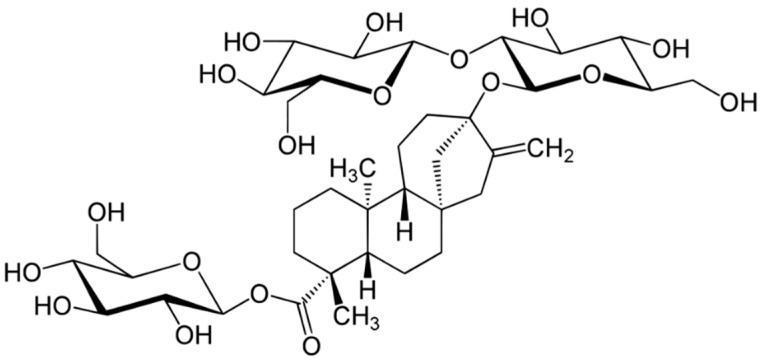
Structural formula of stevioside.

In a zebrafish model of diet-induced obesity, stevioside (a glycoside derived from an extract of plants of the genus Stevia, Figure 7) has been shown to improve glucose tolerance, oxidative stress, and inflammatory mediators linking obesity to insulin resistance, as well as its epigenetic regulation [85].

Stevioside therapy increases the ability of diabetic rats to tolerate glucose and insulin and returns abnormally high levels of glucose, serum insulin, and lipid profile to normal. Stevioside contributes to the normalization of altered levels of lipid peroxidase, hydrogen peroxide and hydroxyl radical, antioxidant enzymes, and insulin signaling molecules, including the insulin receptor, insulin receptor-1 substrate, and Akt mRNA levels. In addition, stevioside enhanced glucose uptake and oxidation in diabetic muscles by very effectively increasing the synthesis of glucose transporter 4, similar to metformin [87]. Stevioside supplementation also increased total calorie intake while decreasing BMI, waist circumference, waist-to-hip ratio, and fat mass index in the obese group [88].

## 5. Mechanisms of Action of Medicinal Plant Components on the Course of DM

Currently, 150–200 species of medicinal plants with hypoglycemic effects are used in medical procedures. Along with food ingredients (proteins, fats, carbohydrates), plants also contain biologically active substances, among which hypoglycemic compounds (galenin, inosine, inulin) play a leading role [34].

According to some reports, phytopreparations contribute to the restoration of insulin production by pancreatic beta cells. Some medicinal plants (ginseng, eleutherococcus, oplopanax, etc.) have an immunostimulating effect, normalizing disorders specific to diabetes mellitus. These and other herbal remedies boost both the central and automatic nervous systems. By stimulating the vagoinsular nervous system, phytopreparations increase the function of the pancreas. Many plants, due to the content of their highly active substances, provide anti-inflammatory, choleretic, sedative, and tonic effects and enrich the body with vitamins and microelements, favorably affecting carbohydrate and other types of metabolism, increasing the overall resistance of the body [47].

One cannot anticipate a rapid therapeutic effect given the low concentration of active components in plants. Phytotherapy should be practiced for a long time, observing medicinal preparation technology and evaluating the effectiveness of their effect on well-being, carbohydrate metabolism, and other indicators. In the case of insufficient effect, treatment tactics must be reconsidered in order to achieve a positive effect [50].

**Figure 8:**
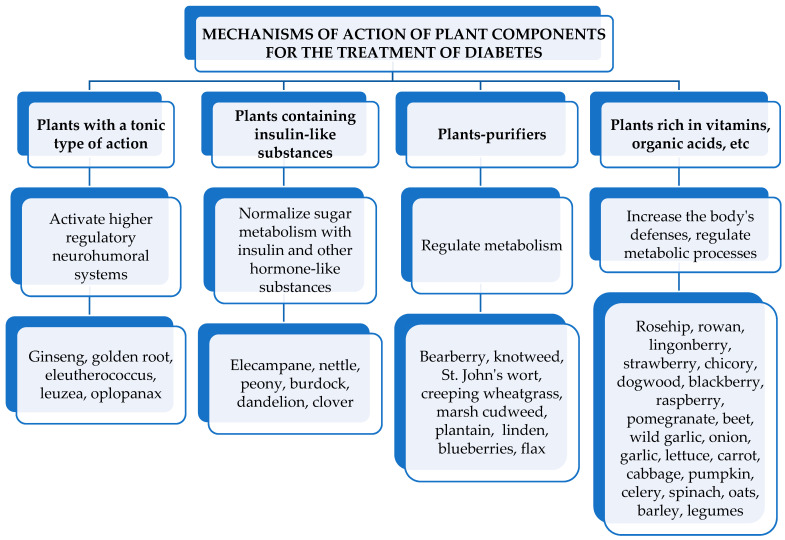
Mechanism of action of medicinal plants in diabetes mellitus.

More frequently than others, the references mention the following plants as having a positive effect on the course of diabetes: white birch, redhaw hawthorn, lingonberry, black elderberry, speedwell, galega (goat’s-rue), common knotgrass, St. John’s wort, wild strawberry, common nettle, corn silk, laurel, flax, greater burdock, lady’s mantle, common juniper, peppermint, comfrey, water dropworts, walnut, broadleaf plantain, lilac, black currant, licorice, marsh cudweed, common beans, chicory, thyme, blueberries, mulberries, and rose hip [36,39]. It would be incorrect to believe that herbal treatment for diabetes is limited only to insulin-like action. Many plants can perform other diverse and useful functions in the body [36]. A generalized diagram representing the mechanism of action of medicinal plants in diabetes mellitus is presented in Figure 8.

The above classification is conditional, since some plants can be assigned to several groups [36]. Nonetheless, it enables the maneuvering of herbal remedies, the selection of appropriate remedies for specific patients and their individual manifestations of a disease and replacing plants in case of intolerance. Furthermore, this classification enables us to approach a logical combination of plant substances. The choice of medicinal plants that have a hypoglycemic effect depends on the type of diabetes mellitus and the presence of concomitant symptoms. Phytotherapy is used to lower blood sugar, regulate metabolism, and prevent the elimination of disease-related symptoms when used as directed and under the supervision of an endocrinologist [39].

## 6. Groups of Biologically Active Substances of Medicinal Plants Used for the Treatment of DM

### 6.1. Insulin-like Substances of Plants, Their Mechanism of Lowering Blood Sugar

Insulin is a protein hormone responsible for carbohydrate metabolism regulation. The physiological effects of insulin are manifested due to its binding to the insulin receptor on the plasma membrane of target cells [89]. Insulin signaling in target tissues causes a variety of biological effects. These events are necessary for normal growth and development, as well as for normal homeostasis of glucose, fat and protein metabolism. The mechanism of action of insulin is a series of cascade signals caused by insulin binding to the insulin receptor. This results in receptor autophosphorylation and tyrosine kinase activation, resulting in tyrosine phosphorylation of insulin receptor substrates. Phosphorylation of insulin receptor substrates leads to the activation of phosphatidylinositol 3-kinase and subsequently to the activation of Akt kinase and its downstream mediator AS160, all of which are important steps in the stimulation of insulin-induced glucose transport. It is believed that impaired activation of phosphatidylinositol-3-kinase and the downstream signaling cascade underlies the emerging insulin resistance [90]. The resulting reduced ability of peripheral target tissues to respond to insulin stimulation leads to insulin resistance [91].

Bioactive plant derived proteins and peptides control type 2 DM by acting on the insulin signaling pathway. Three peptides, aglycine, viglycine, and ILP, act as insulin mimetics (agonists) to stimulate insulin-sensitive target tissues. Therefore, plant derived bioactive proteins and peptides act through biochemical pathways to modulate insulin resistance and the hyperglycemic state [92].

Moringa (*Moringa oleifera)* is a tree native to India. The insulin-like proteins of this plant have hypoglycemic activity [93]. Soy’s insulin-like proteins are of particular interest to researchers. Aglycine, a 37 amino acid bioactive peptide isolated from soybeans, is resistant to digestive enzymes and has antidiabetic properties. Oral aglycine administration could potentially reduce or prevent hyperglycemia by increasing insulin receptor signaling in the skeletal muscle of mice with streptozotocin-induced diabetes/high-fat diet [94]. Aglycine is resistant to pepsin, trypsin, and Glu-C protease in vitro proteolysis, which is consistent with its intestinal origin and exogenous origin from plant foods. When administered subcutaneously to mice (at a dose of 10 μg·g^−1^ of body weight) aglycine has a hyperglycemic effect, leading to a doubling of blood glucose levels within 60 min [95]. Furthermore, in vitro experiments have revealed that the IC50 value of aglycine peptide for α-glucosidase inhibition was lower than that of acarbose, indicating that aglycine peptide exhibits inhibitory activity against α-glucosidase. This further confirms the potential of the aglycine peptide as an α-glucosidase inhibitor [96].

*Bauhinia variegata* L. is a flowering plant in the Fabaceae family whose range extends from China through southeast Asia to the Indian subcontinent. The insulin-like proteins of this plant can lower blood glucose levels after three days of therapy [97].

### 6.2. Plant Antioxidants and Their Antidiabetic Properties

**Figure 9:**
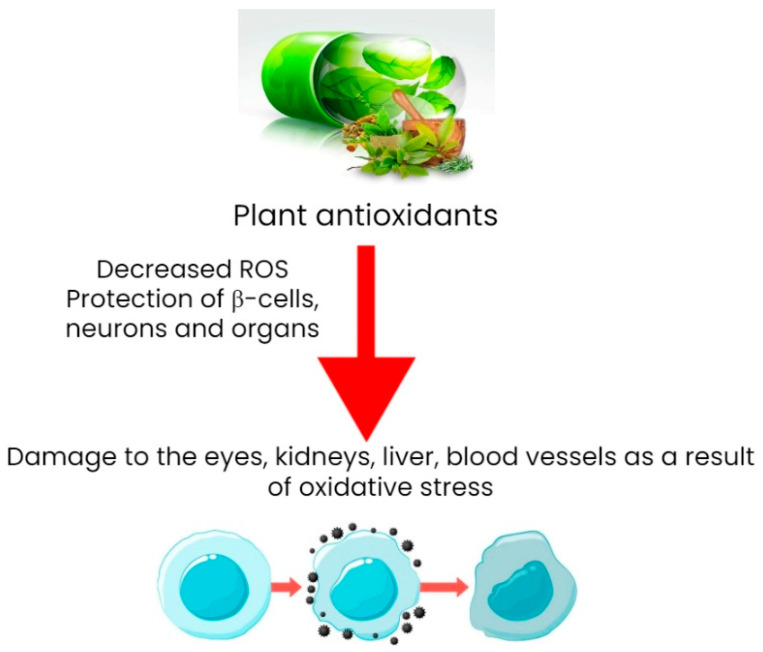
Mechanism of antidiabetic action of plant antioxidants.

Antioxidants are known to reduce the risk of type 2 diabetes [98,99]. It has been confirmed that oxidative stress is one of the most important factors in the pathogenesis of diabetic retinopathy. Hyperglycemia-induced metabolic disturbances, such as the increased flows of the polyol and hexosamine pathways, hyperactivation of protein kinase C (PKC) isoforms, and the accumulation of advanced glycation end products, can all be caused by oxidative stress (age). Moreover, repression of the antioxidant defense system through hyperglycemia-mediated epigenetic modification also leads to an imbalance between ROS clearance and production. Excessive accumulation of ROS causes damage to mitochondria, cellular apoptosis, inflammation, lipid peroxidation, and structural and functional changes in the retina [100,101]. Figure 9 demonstrates the mechanism of the protective action of plants against diabetes.

Numerous studies have been conducted to investigate the role of antioxidants in diabetes [102]. Astaxanthin is a powerful antioxidant found primarily in marine organisms. Several studies in recent years have suggested that astaxanthin may be useful in the treatment of diabetes [103]. It has been shown to protect β-cells, neurons, and several organs, such as the eyes, kidneys, and liver, from the oxidative damage caused by diabetes. Furthermore, it enhances glucose and lipid metabolism, as well as cardiovascular health. Its beneficial effects are manifested through multiple effects on cellular functions [104].

Various epidemiological, clinical, in vivo and in vitro studies have shown that fruits, berries and vegetables, e.g., bitter melon [105,106], passion fruit [107], blackberry [108], peach [109], banana passion fruit [110], snake fruit [111], currants [112], papaya [113], tomatoes [114], capsicum [115], celery [116], onions [117], and others, with different phytochemical profiles can reduce glucose levels in the blood to normal levels. Bioactive compounds with antidiabetic activity include phenolic compounds, polysaccharides, and vitamin C, and most of them have antioxidant activity or can induce antioxidant systems in various experimental models [118,119].

*Vernonia amygdalina* (bitter leaf) is used as a food ingredient and also as a supplement in the treatment of type 2 diabetes (DM 2). It is a potential antidiabetic agent due to its ability to inhibit intestinal glucose absorption, increase muscle glucose uptake, and protect the liver from oxidative stress. The ability of the infusion to reduce oxidative stress, DNA fragmentation, and stimulate glucose uptake by muscles may indicate that *V. amygdalina* has antioxidant, anti-apoptotic, and insulin-sensitizing activity [120].

Plant antioxidants found in green tea, hibiscus sabdariffa, and garlic, such as resveratrol, quercetin, curcumin, ferulic acid, and phloretin, can inhibit NF-κB (a nuclear factor enhancer of activated B-cell κ-light chains) activation and reduce the expression of target genes, including those involved in inflammation [121]. Polyphenols found in sweetgale (*Myrica gale* L.), cinnamon rose (*Rosa majalis*), sorrel (*Rumex acetosa* L.), nettle (*Utrica dioica* L.), and dandelion (*Taraxacum officinale* L.) can be used to treat diabetes type 2 and obesity, because they can scavenge free radicals and subsequently inhibit α-amylase, α-glucosidase, and advanced glycation end products [122].

### 6.3. Plant Polysaccharides with Antidiabetic Properties

Plant and fungi polysaccharides are now recognized as potent pharmacological agents with significant therapeutic potential [123]. Plant polysaccharides are extremely safe and have a wide range of pharmacological activities, including immunoregulatory, antitumor, antioxidant, anti-aging, and other properties. Recent research has shown that many polysaccharides are beneficial in treating metabolic diseases such as cardiovascular disease, diabetes, obesity, and neurological diseases, which are typically brought on by impaired fat, sugar, and protein metabolism [124]. Dietary polysaccharides are mostly derived from natural sources, such as plants, fungi, algae, etc. They are resistant to human digestion and absorption, and ferment completely or partially in the colon [125,126].

**Figure 10:**
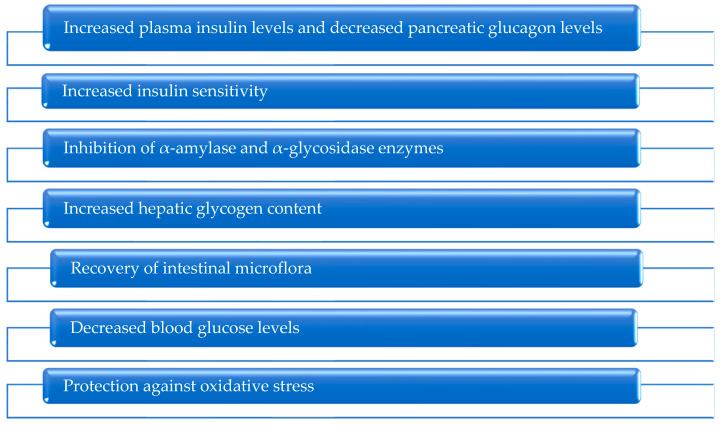
Main antidiabetic mechanisms of action of polysaccharides.

The mechanisms of their action are associated with the regulation of apoptotic, inflammatory, oxidative stress, intestinal microbiota, and many metabolic pathways (Figure 4). Because polysaccharides are a natural product, they can be used as fat or sugar substitutes [124]. Figure 10 depicts the main mechanisms of polysaccharide antidiabetic action, with examples of these mechanisms discussed below.

#### 6.3.1. Increased Insulin Levels and Decreased Pancreatic Glucagon Levels

A polysaccharide isolated from *Dendrobium officinale* (family *Orchidaceae*) has antidiabetic activity, which is likely due to the regulation of glucagon-mediated hepatic glycogen metabolism and gluconeogenesis, as well as hepatic glycogen structure [127]. Polysaccharides from the stems of this plant increase the level of insulin and glucagon-like peptide-1 [128].

#### 6.3.2. Increased Insulin Sensitivity

Polysaccharides are extracted from various parts of *Anoectochilus roxburghii* and *Anoectochilus formosanus* (Orchidaceae family) plants, which exhibit various antidiabetic activity by increasing insulin sensitivity, inhibiting hepatic gluconeogenesis, and lowering triglyceride levels and low-density lipoprotein cholesterol [122]. Polysaccharides from *Enteromorpha prolifera* (green algae of the *Ulvaceae* family) promote insulin sensitivity by activating the PGC-1α-FNDC5/irisin pathway [129]. In addition, polysaccharides can protect damaged pancreatic islets in mice [130].

The aim [17] was to clarify some mechanisms of radical scavenging and the presence of biological activities of high molecular weight fucoidan, from *Fucus vesiculosus,* that are anti-inflammatory, antihyperglycemic, and anticoagulant. Fucoidan showed strong activity in removing 1,1-diphenyl-2-picrylhydrazyl-hydrazyl radicals and reducing activity. It significantly inhibited the enzyme cyclooxygenase-2 (COX-2) (IC_50_ 4.3 µg/mL) with a higher selectivity index (Ig(IC_80_ COX-2/IC_80_ COX-1), −1.55) than the synthetic non-steroidal anti-inflammatory drug indomethacin (Ig (IC_80_ COX-2/IC_80_ COX-1), −0.09). Concentration-dependent inhibition of the hyaluronidase enzyme was observed with an IC_50_ of 2.9 µg/mL. Fucoidan attenuated lipopolysaccharide-induced expression of mitogen-activated protein kinase p38. The data obtained suggest that inhibition of dipeptidyl peptidase-IV (DPP-IV) (IC_50_ 1.11 μg/mL) was one of the possible mechanisms for the antihyperglycemic activity of fucoidan. At a concentration of 3.2 μg/mL, fucoidan lengthened the activated partial thromboplastin and thrombin time by 1.5 and 2.5 times, respectively, compared with the control. A significant increase in prothrombin time was observed after increasing the concentration of fucoidan above 80 µg/mL. This suggests that fucoidan may influence internal/common pathways and have little effect on extrinsic pathways. This study sheds light on the multiple pathways of fucoidan’s biological activity. To the best of our knowledge, the inhibition of hyaluronidase and DPP-IV by high molecular weight fucoidan was studied for the first time in this study [17]. The presented results and literature data suggest that molecular weight, sulfate content, fucose content, and polyphenols may contribute to this activity. High molecular weight fucoidan appears to have promising therapeutic applications in a variety of pharmacological settings.

#### 6.3.3. Inhibition of α-Amylase and α-Glycosidase Enzymes

Many non-starch polysaccharides of plants have inhibitory effects on type 2 DM-associated enzymes [131]. Polysaccharides isolated from *Aconite coreanum*, one of the *Aconite* species, show inhibitory activity against the glycosidase enzyme, preventing glucose from entering the bloodstream quickly [132]. Polysaccharides from bitter melon (*Momordica charantia* L.) and raw garlic bulbs (*Allium sativum* L.) have strong antioxidant properties and show inhibitory activity against α-amylase and α-glycosidase [133,134]. Guava leaves (*Psidium guajava* L., *Myrtaceae*) have long been used in Asia and North America as a folk herbal tea for diabetes. The polysaccharides of this plant can scavenge free radicals, and also significantly reduce fasting blood sugar levels by inhibiting the enzymes α-amylase and α-glucosidase [135].

#### 6.3.4. Increased Hepatic Glycogen Content

The combination of inulin and *Ganoderma lucidum* polysaccharides promotes the synthesis of glycogen, a polysaccharide that serves as the main form of glucose storage [136]. The polysaccharides of the brown algae *Undaria pinnatifida* can protect pancreatic islet cells from damage while stimulating glycogen synthesis in the liver [137].

#### 6.3.5. Normalized Intestinal Microflora

Polysaccharides regulate the intestinal flora, improve glucose and lipid metabolism disorders, maintain the balance of the islet internal environment, and reduce systemic inflammation [138]. Some water-soluble non-starch polysaccharides of cereals, such as oats, glucans, and guar gum, have been reported to reduce glucose absorption, the rate of gastric emptying—and thus the postprandial increase in blood sugar levels—and insulin levels, both in healthy people and diabetic patients, due to their ability to increase viscosity in the gastrointestinal tract [139]. Coix seed polysaccharides (CSP) have a hypoglycemic effect through the gut. The use of these seeds in therapy alters the microbial composition of the intestine, particularly bacteria that produce short-chain fatty acids, activating the IGF1/PI3K/AKT signaling pathways and exhibiting hypoglycemic activity [77]. In streptozotocin-induced diabetic rats, oral administration of Momordica charantia polysaccharides resulted in a significant normalization of kidney function test parameters [140]. Mushroom polysaccharides also act as prebiotics and modulate gut microflora composition and thus may reduce insulin resistance [141].

#### 6.3.6. Decreased Blood Glucose Levels

A polysaccharide known as β-glucan, which is found in yeast, fungi, bacteria, algae, barley, and oats [142], may aid in the regulation of glycemic responses. Numerous factors, including the nature of the food and the concentration and molecular weight of β-glucan, have been found to influence such interactions. Among all these, the dose of β-glucan is considered the most important factor regulating the effect of fiber on glycemic responses. Breakfasts containing 4.6 or 8.6 g of β-glucan have been shown in studies to significantly lower mean serum insulin and glucose concentrations when compared with non-insulin dependent diabetic subjects. It was observed that the content of exogenous glucose is 18% less in polenta flour containing oat β-glucan (5 g) compared with control polenta flour without oat β-glucans [143].

#### 6.3.7. Oxidative Stress Protection

According to some researchers, the antidiabetic effects of polysaccharides are primarily due to their antioxidant properties. The antioxidant activity of polysaccharides helps to reduce the degree of damage to β-cells in the pancreas [144]. Pumpkin polysaccharides have antioxidant, antitumor, immunoregulatory, hypoglycemic, and hepatoprotective activity. Ji et al. have shown that lower molecular weight uronic acid-rich pumpkin polysaccharides tend to have increased biological activity, while the (1 → 3) and (1 → 4)-Glcp glycosidic linkages in the main chains have hypoglycemic effects [145].

### 6.4. Plant Alkaloids with Antidiabetic Properties

Alkaloids are a class of naturally occurring chemical compounds derived from plants, animals, bacteria, and fungi. They have a wide range of pharmacological activities such as antimalarial, antiasthma, anticancer, antihypertensive, oxytotic, CNS stimulant, muscle relaxant, antispasmodic, cholinomimetic, vasodilator, antiarrhythmic, analgesic, antibacterial, and antihyperglycemic. Several alkaloids, including berberine, boldine, and sanguinarine, have been demonstrated to be potentially effective against various diabetes models [71].

The interaction of alkaloids with a variety of proteins involved in glucose homeostasis is the mechanism underlying their antidiabetic effects. Each class of alkaloids has two or more biological activities in which they act as antidiabetic metabolites [146].

Medicinal species such as capsicum (*Capsicum annuum*), turmeric (*Curcuma longa*), barberry (*Berberis vulgaris*), and garden cress (*Lepidium sativum*) are among the most common and therapeutic plants used to control diabetes and have been the subject of several experimental and clinical studies. Alkaloids isolated from these plants (berberine, capsaicin, and trigonelline) are of great interest in this area. Interestingly, the therapeutic effect of alkaloids on blood glucose pathogenesis is mediated through various signaling cascades and pathways, such as inhibition of the α-glucosidase enzyme, blockade of PTP-1B, deactivation of DPP-IV, increased insulin sensitivity, and oxidative stress modulation [147]. Additionally, alkaloids can inhibit the enzymes α-amylase, α-glucosidase, aldose reductase, dipeptidyl peptidase-IV and protein tyrosine phosphatase-1B; inhibit the end products of glycation; increase insulin secretion; and enhance glucose uptake [148].

Eurocristatin (ECT) is an alkaloid isolated from Eurotium cristatum that improves glucose metabolism and alleviates insulin resistance by activating the PI3K/AKT signaling pathways [149,150].

## 7. Complications of DM and the Effect of Medicinal Plants and Their Phytocomponents on Them

The absolute or relative deficiencies of insulin and insulin resistance contribute to the development of various metabolic and vascular diseases, neuropathies, and pathological changes in internal organs and tissues, including the digestive system [151,152,153]. The diabetic syndrome is characterized mainly by lesions of the lower extremities. The main pathogenetic factors leading to the development of diabetic foot are peripheral nephropathy and damage to the large arteries of the lower extremities, leading to infection [154,155]. A decisive role in the development of diabetic retinopathy is played by chronic hypoglycemia and associated biochemical disorders (formation of sorbitol, non-enzymatic glycosylation of retinal vascular proteins, increased oxidative stress).

Due to their antioxidant and membrane stabilizing effects, flavonoids can reduce vascular wall permeability and inflammation, as well as determine the antioxidant, anti-inflammatory, and diuretic effects of preparations containing these substances [156]. Polyphenolic compounds in phytocomponents react with free radicals to form less active phenolic radicals, facilitating the utilization of oxidized sugars and rapidly slowing the sugar oxidation process in the body. The inhibitory effect of preparations stabilizes the structure of cell membranes, normalizes permeability, improves microcirculation and accelerates the utilization of toxic substances. The end result is the prevention of severe organ damage and the activation of regenerative processes [157].

The blueberry, a member of the lingonberry family, is a plant that may help to reduce the side effects of DM. It contains tannins, myrtilene, a mixture of delphidin monomethyl ether and malvidin chloride, vitamins C, B, and carotenes, and it has recently been used to treat diabetes. Neomyrtilene in the leaves of the plant significantly reduces blood glucose levels in experimental diabetic patients [158,159].

Soy contains flavonoids, amino acids, beta-carotene, and vitamins E, B, and C. Studies have shown that soy extract dissolved in water reduces blood sugar levels by 30–40%, has a diuretic effect and improves pancreatic function [160]. This makes the use of soy in DM highly beneficial, in addition to its use as a diuretic and renal drug [157]. Vitamin K, uric acid glycosides, formic acid, tannins and proteins, vitamins C and B2, trace elements, flavonoids, chlorophyll, and carotenoids are all present in fresh nettle leaves [161]. Kuril tea extract has an anti-inflammatory effect, manifested by a decrease in blood sugar and lipid levels, a protective effect against diabetes, and functional activity of the liver and kidneys. The plant’s therapeutic effects on experimental diabetes have been tested on laboratory animals and it contains flavonoids, vitamin C, carotenes, and tannins. It has been established that it reduces the degree of damage to the islets of Langerhans, slows down the development of diabetes and hypoglycemia, and increases resistance to the toxic effects of DM. Dandelion is an insulin-containing plant that increases the activity of the pancreas, increases insulin secretion, and improves digestion and metabolism [162]. Fennel root contains tryptophan compounds, sterols, and 24% of insulin. It is customary to collect the rhizomes along with the aerial parts of the plant for medicinal purposes.

The use of plant extracts and phytochemicals is currently popular for the prevention or treatment of various health problems; though this creates problems when classifying them as dietary supplements or nutraceuticals, because they do not require proof of effectiveness [125]. The use of herbal medicines or herbal ingredients in combination with traditional medicines requires product approval, including safety measures, quality control, and efficacy data [163].

The bioavailability of a plant extract or plant component is critical to its full effect on the body and includes the steps of delivery, absorption, distribution, metabolism, and clearance of the extract/component. The crude plant extracts or plant components showed good biological activity (such as antioxidant activity) in vitro, and a slight decrease in activity was observed in in vivo studies. One of the main reasons why plant extracts or plant compounds work more effectively in vitro is the use of higher effective concentrations than those commonly used in in vivo studies. When used in vivo, effective concentrations reach target tissues or organs after absorption, distribution, metabolism, and degradation, and exhibit biological responses at concentrations lower than those tested in vitro [164]. One of the main trends in the research and application of herbal medicine in recent decades is the strategy of increasing the biological activity of biologically active compounds from plant extracts by isolating the pure components of the extract. Several studies have shown that many biologically active ingredients have reduced pharmacological efficacy compared with untreated biomaterials. Some ingredients (mainly primary and secondary plant metabolites) boost biological activity in the body or regenerate bioactive components (including endogenous natural nanoparticles), making raw extracts biocompatible. Testing the bioavailability of plant extracts or phytochemicals is important when determining potential health benefits, but also helps evaluate unwanted toxic side effects, including establishing the concentration/dose of exposure at which side effects occur. Efforts have been made to harmonize toxicity testing methods for various plant products intended for human consumption [165,166]. Toxicity research includes studies on low dose acute toxicity, high dose acute toxicity, and specific cellular, organic, and systemic toxicity.

In general, all studies of plant extracts for disease prevention and treatment are based on the variety of herbal remedies studied, the time of absorption by the human body, therapeutic doses reaching the target tissue, their ability to bind to other drugs, and factors such as pharmacological or medical parameters of plant extracts and bioactive compounds, age, gender, and population health status [166].

## 8. Current Progress and Prospects for the Use of Medicinal Plants and Phytocomponents in the Treatment of Diabetes

Patel et al. [167] have reported many medicinal plants with hypoglycemic potential that belong to various families when examining the advances and prospects of medicinal plants and their phytocomponents in the treatment of diabetes (Fabaceae, Lamiaceae, Liliaceae, Cucurbitaceae, Asteraceae, Morasaceae, Rosaceae, and Araliaceae), with *Allium sativum, Gymnema sylvestre, Citrullus colocynthis, Trigonella foenum-graecum, Momordica charantia,* and *Ficus benghalensis* holding their particular interest. Some new biologically active compounds isolated from plants such as roseoside, epigallocatechin gallate, beta-pyrazol-1-ylalanine, cinchonain-Ib, leukocyanidin 3-O-β-d-galactosyl cellobioside, leukopelargonidin-3-O-α-L, rhamnoside, glycyrrhetinic acid, etc., have proven to be more effective than conventional hypoglycemic agents.

A number of in vivo studies in model systems, such as mice, rats, rabbits, and humans, have shown that complex plant components have a positive effect on reducing the risk of hypercholesterolemia as well as improving the treatment of this disorder [168,169]. The consumption of legume starches can have a positive effect on glycemia due to a persistent effect on postprandial glycemia without a sharp increase. In addition, it can prevent both postprandial hyperglycemia and late hypoglycemia [170]. Plant polysaccharides increase serum insulin levels by several mechanisms, lowering blood glucose levels, and improving glucose tolerance. Many phytochemicals have been reported to have beneficial effects in the treatment of hyperglycemia [171]. Some examples of the antidiabetic action of phytocomponents are presented in Table 1.

Since oxidative processes are the main cause of some metabolic diseases and age-related degenerative disorders, herbs and spices as sources of antioxidants are of great interest for the treatment of many diseases, such as diabetes [175]. In addition, various vitamins and minerals play an important role in the treatment of diabetes. For example, vitamin C (ascorbic acid) is a pre-eminent antioxidant that participates in several non-enzymatic reactions. Moreover, it is an electron donor, effectively scavenging free radicals and inhibiting lipid peroxidation and also promotes the regeneration of vitamin E and reduced glutathione [176]. In animals, vitamin C reduces the accumulation of sorbitol and lipid peroxides in erythrocytes induced by diabetes [177].

A daily dose of 1000 mg of vitamin C may help prevent or reduce the development of cataracts and nerve disorders, which are serious complications of diabetes. Furthermore, it inhibits protein glycosylation, which is linked to the development of long-term diabetes complications. Given the role of oxidative stress in the pathophysiology of DM and especially in the pathogenesis of β-cell dysfunction, antioxidant compounds extracted from plants may be useful in the treatment of diabetes and its complications [178].

Vanadium, found in all cells, acts as an insulin mimetic. It is found in mushrooms, shellfish, black pepper, parsley, dill seeds, beer, wine, and cereals. According to animal and in vitro studies, vanadium has an insulin-like effect on the liver, skeletal muscle, and adipose tissue [179]. Moreover, it stimulates glucose uptake (directly or by inhibiting the phosphotyrosine phosphatase enzyme system), thereby enhancing insulin receptor phosphorylation and insulin receptor (IR) interaction with tyrosine kinase [180].

Abundant in some plants, such as sunflower and leuzea, ω-3 fatty acids have been reported to improve insulin resistance in animal models [181]. It is known that, despite the indirect influence on the mechanisms of diabetes, magnesium interferes with the protection against complications of diabetes and has a recommended daily dose of 1000 mg/day. Additional approaches, such as the use of medicinal plants, herbs, and/or formulations and ω-3 polyunsaturated fatty acids (PUFAs) with hypoglycemic and lipid-lowering activity, can be used as alternatives to oral hypoglycemic agents (OHA), which are known for their side effects [182].

All of the data presented demonstrate the therapeutic potential of plants with anti-diabetic properties and their components that can be used as nutraceuticals to alleviate the symptoms of diabetes and improve quality of life. However, this strategy depends on many parameters, such as safety, long-term side effects, and toxicity, as well as supplementation studies and human clinical trials, to prove the necessary positive effects on human health [183].

## 9. Conclusions

This review considers the main plants used for the treatment of diabetes mellitus and the mechanisms of action of their main components for the first time. The review is the first to examine the main secondary metabolites of plants that have anti-diabetic properties, including insulin-like proteins and peptides, polysaccharides, antioxidants, and alkaloids. It has been established that drug metabolites in diabetes mellitus have various therapeutic mechanisms. Plants with a general tonic effect, plants containing insulin-like substances, plant purifiers, and plants rich in vitamins, organic acids, and other nutrients have been shown to play an important role in the treatment and prevention of type 2 diabetes mellitus and its complications.

Extensive antidiabetic drug screening research over the last several decades has established natural products as a major potential source of drug discovery. However, only a few herbal preparations have received scientific confirmation. Polysaccharides, antioxidants, alkaloids, and other plant compounds have proven antidiabetic effects. The antidiabetic effects of active plant compounds can be seen both in their combined effects and in the use of each compound alone. The antidiabetic properties of medicinal plants can also be attributed to secondary metabolites such as phenolic acids, glycosides, saponins, stilbenes, tannins, and others. Antidiabetic activity can be influenced by plant growth conditions, methods of collection, drying and storage, and methods of extraction and purification of active compounds. Therefore, a thorough analysis of how these factors affect the antidiabetic potential of end products is required. The search for new sources of potentially inexpensive plant materials that do not require special growth conditions will simplify the introduction of plant-derived compounds into the pharmaceutical industry.

## Figures and Tables

**Table 1:** Examples of antidiabetic action of phytocomponents.

Plant	Phytocomponents	Advantages	Disadvantages	Source
*Dendrobium officinale*	Polysaccharides	↑ Glycogen synthesis ↓ Gluconeogenesis	↓ Fatty acid synthesis	[172]
*Anoectochilus roxburghii*	Polysaccharides	↓ Liver lipids ↑ Liver sensitivity to insulin	↑ Fatty acid oxidation ↓ Glucose disposal by liver	[173]
*Capsicum annuum*	Alkaloids	↑ Energy consumption ↓ Blood glucose ↑ Insulin sensitivity ↓ Serum lipids	↑ Fatty acid oxidation	[170]
*Costus igneus*	Proteins/peptides	↓ Fat intake, fatty acid synthesis ↑ Energy consumption	↑ Fatty acid oxidation	[168]
*Glycine max*	Peptides	↑ Adipocyte differentiation	↑ Fat accumulation	[156]
*Myrica gale* L.	Polyphenols	↓ Blood glucose ↑ Sensitivity to insulin	↑ Glucose absorption	[174]
*Vernonia amygdalina*	Antioxidants	↑ Glucose clearance	↓ Glucose disposal by liver	[33]
*Rosa majalis*	Antioxidants	↑ β-cell regeneration ↓ Glucose absorption	↓ α- amylase and α- glucosidase	[25]

↑ or ↓—increase or decrease, respectively.
